# Single-Stage Reconstruction of a Devastating Antebrachial Injury With Brachial Artery, Median Nerve, and Soft Tissue Deficit: A Case Report and Review of the Literature

**Published:** 2010-04-30

**Authors:** Kyle A. Belek, Lee W. T. Alkureishi, Ashley A. Dunn, Zlatko Devcic, Mauricio Kuri, Charles K. Lee, Scott L. Hansen

**Affiliations:** Division of Plastic Surgery, Department of Surgery, University of California, San Francisco, CA 94143

## Abstract

**Objective:** We present a case of a 31-year-old man who fell through a skylight sustaining a deep laceration injury to his dominant arm. A single-stage radial artery flow-through free flap and cabled sural nerve graft for reconstruction of a complex antebrachial defect involving skin, soft tissue, muscle, brachial artery, and median nerve was performed. A technical description of the case and review of the literature are described. **Methods:** Traumatic injuries to the arm and antecubital fossa often lead to devastating outcomes. Advances in microsurgical technique as well as improved skin and dermal substitutes have allowed improved outcomes as well as shorter hospital stays. In this case, surgical treatment involved microsurgical reconstruction of the brachial artery with a radial artery flow-through flap and a single-stage donor-site closure with an Integra dermal matrix template and split-thickness skin graft. **Results:** Successful vascular flow and soft tissue coverage were performed with successful salvage of the limb. **Conclusion:** A single-stage reconstruction versus a multistage, delayed reconstruction of a devastating arm injury with a radial forearm flow-through flap and single-stage closure with Integra and autologous skin graft can provide a safe, effective, and clinically satisfactory outcome.

Traumatic injuries to the arm and antecubital fossa often lead to devastating outcomes. Advances in microsurgical technique as well as improved skin and dermal substitutes have allowed improved results as well as decreased overall cost and shorter hospital stays. As microsurgical techniques have improved, so has the ability to repair these injuries in a single stage with satisfactory clinical outcomes as well as to definitively close these wounds with dermal substitutes such as Integra. We present the case of a large soft tissue deficit accompanied by a brachial artery and median nerve defect. Microsurgical reconstruction and coverage of the lesion as well as a review of the literature are described.

## METHODS

### Case example and description of surgical technique

**Figure 1:**
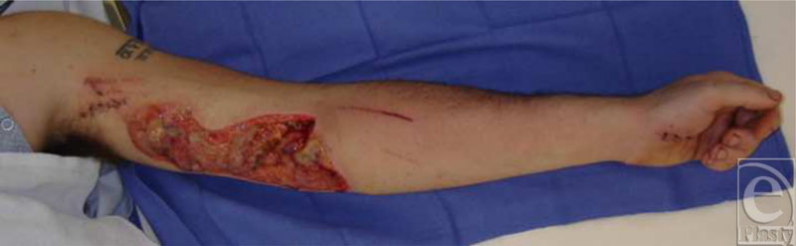
Pre-operative view.

**Figure 2:**
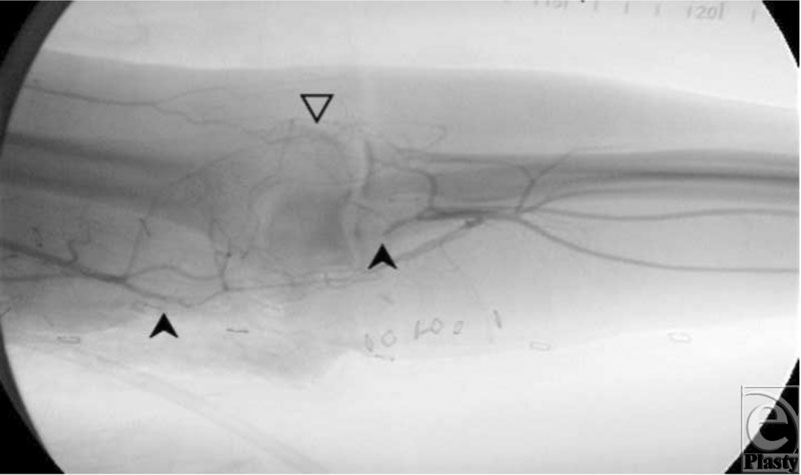
Solid arrows: brachial artery. Open arrow: collateral vessels.

**Figure 3:**
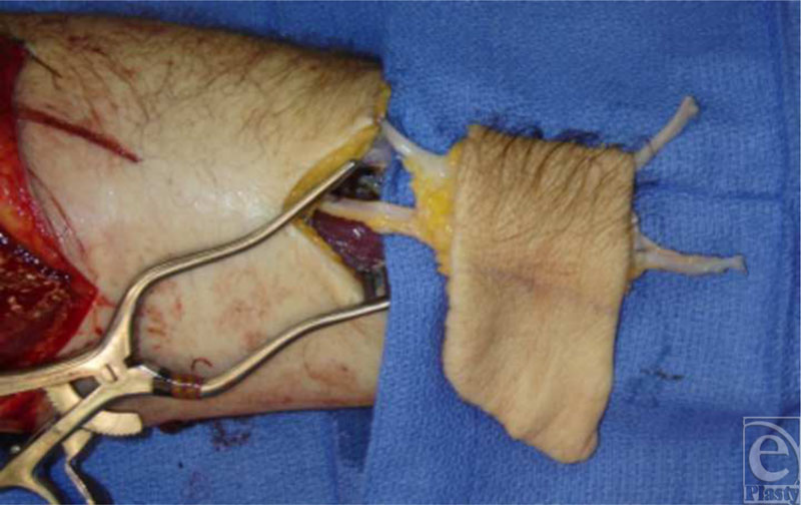
Intra-operative view.

**Figure 4:**
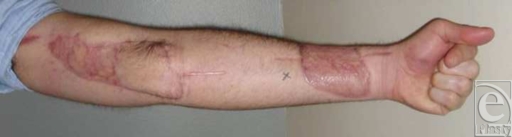
Post-operative view.

A 31-year-old man suffered an injury to his dominant left arm after falling through a skylight. Following initial resuscitation and operative hemostasis, the skin and muscle defect stretched from the level of the mid-humerus to the anteromedial elbow and measured 250 cm^2^ (Figure 1). Segmental loss of the brachial artery (8 cm) and the median nerve (7 cm) was also evident (Figure 2). An angiogram was obtained, which demonstrated adequate collateral circulation. Following continued exploration and debridement, a radial forearm flow-through free flap was raised from the ipsilateral side. The sural nerve was harvested, and 3 cabled nerve grafts were used to bridge the median nerve defect (Figure 3). The radial artery was interposed between the proximal and distal ends of the brachial artery, and the venae comitantes was anastomosed to the brachial vein. Vascular inflow to the hand was restored and the remainder of the flap was inset. The donor site was definitively closed with a split-thickness skin graft and Integra during the operation while a portion of the recipient site required a split-thickness skin graft (Figure 4). A negative pressure wound therapy (NPWT) device (EZ Care; Smith & Nephew, Largo, Fla) was used for dressing the donor and recipient sites.

## RESULTS

The patient is now 6 months postoperative. He had no immediate postoperative complications. His donor and recipient sites have healed without the need for a second-stage excision and grafting. The patient's hand remains well perfused, with protective sensation in the median nerve distribution. He has a positive Tinel's sign at the mid-forearm and absence of anterior interosseus nerve function. He is currently undergoing physical therapy.

## LITERATURE REVIEW

Case reports have provided multiple options for repair of complex upper extremity injuries. Despite the method of reconstruction chosen, we have universally noted that the authors address the importance of early repair as well as treatment in a center that specializes in microsurgical care. The use of the radial forearm free flow-through flap provides autologous soft tissue coverage as well as native vasculature to reestablish inflow to the involved extremity. To expand upon the review by Bullocks et al,^1^ we discuss the history of flow-through flaps and further discuss the evolution of artificial soft tissue substitutes.

In 1983, Soutar et al^2^ first described a radial artery flow-through flap to anastomose the external carotid artery to the facial artery for a head and neck reconstruction. Foucher et al,^3^ just 1 year later in 1984, performed the first radial forearm flow-through flap for an extremity reconstruction with a concomitant vascular defect. In 1991, Costa et al^4^ further addressed the importance of this type of flap with two cases of reconstruction involving the hand and foot. Since that time, flow-through flaps have become a valuable treatment modality for complex reconstructions.

In 1998, Yavuz et al^5^ described the case of a 13-year-old girl who sustained a gunshot to the cubital region that would have likely resulted in amputation. However, with the use of a radial forearm flow-through flap from the near amputated part, they were able to salvage the extremity. In 1999, Kasten et al^6^ described the case of a 28-year-old man who suffered an injury to the soft tissue, median nerve, and brachial artery in his dominant arm after punching a window. A radial forearm flow-through flap was used 1 week postoperatively after a failed saphenous vein reconstruction lead to an ischemic distal extremity. The authors cite the benefits of a single-stage reconstruction that avoids multiple donor sites and also provides an arterial conduit for vascular repair.

In 2007, Kesiktas et al^7^ described a series of 5 patients with wide tissue deficits and segmental defects in the brachial artery. The authors used a radial forearm flow-through flap for several purposes. They cite numerous advantages including working in a single surgical area, shorter dissection times, suitability of vessel caliber and length, as well as offering tissue from the distal aspect of the wound and avoiding additional areas of morbidity.

With regard to artificial dermal closure, in 1981, Burke et al^8^ described a physiologically acceptable artificial skin substitute for the treatment of extensive burn injuries. In 1990, Heimbach et al^9^ reported the first multicenter results demonstrating improved healing time and favorable aesthetic outcomes with the use of artificial dermal grafts. Application of the dermal template required a second operation for the placement of epidermal grafts at approximately 7 days postoperatively. The use of artificial dermis has since evolved to become a commonly used product for burn and reconstructive surgery, and a full background check of its use is beyond the scope of this review.

However, there have been reports in the literature of the application of negative pressure wound dressings placed over the dermal template to assist with wound closure. An article published in 2009 by Leffler et al^10^ reported the case of a 25-year-old man who required a dermal template (Integra) that was covered with a vacuum dressing that lead to complete graft take, avoided the need for dressing changes, and allowed the patient to be treated as an outpatient. Also, in 2009, Burd et al^11^ reported a series of 10 patients who underwent circular excisions for facial cancers that were successfully repaired using a single-stage Integra approach with subsequent successful closure of the epithelial defects with calcium alginate.

To our knowledge, this is the first case of a single-stage closure with placement of Integra and a thin piece of split-thickness skin graft at the original operation with successful closure beneath a NPWT device (EZ Care, Smith & Nephew). In 2007, Kim and Hong^12^ described a similar method by using a single-stage closure of a donor site with Alloderm (Lifecell Corp, Branchburg, NJ) and split-thickness skin graft under NPWT. They found a significantly improved rate of graft take as well as a decreased time to closure. From a technical perspective, it is important to note that both the Integra and the skin graft should be meshed 1.5 to 1 under a protective wound layer such as Acticoat (Smith & Nephew). This meshing allows for continued fluid removal beneath the grafts as well as improved imbibition of the grafts for improved graft take.

## DISCUSSION

Devastating injuries to the antebrachial region involving the soft tissues, vasculature, and nervous structures provide a challenge to the reconstructive surgeon. A review in 1990 describes the incidence of upper extremity vascular injury over 4 years in an urban trauma center. Eighty patients with 123 vascular injuries required surgical care, and the majority of these patients had concurrent nerve, vein, bone, or soft tissue injuries. The authors suggest an aggressive approach to the diagnosis and treatment of complex upper extremity trauma, emphasizing meticulous surgical technique, liberal use of fasciotomy, and aggressive intraoperative debridement and repair of associated injuries.^13^ With advances in microsurgical techniques as well as improved skin substitutes, there has been an improvement in clinical outcome with single-stage procedures, decreased overall cost to the patient and the hospital, as well as shortened hospital stays.^10^

The radial forearm flow-through flap has proven useful for several reasons. Arterial revascularization accompanied with native soft tissue coverage avoids the potential pitfalls of reconstruction with foreign material.^6^ Also, obtaining tissue from the affected extremity allows for operating within a single surgical area and avoiding donor-site morbidity from other regions of the body.^7^ However, the authors recognize the potential pitfalls of obtaining soft tissue with its blood supply in an already vascularly compromised field. The potential for ischemia far outweighs any benefit that we may justify by limiting donor-site morbidity in the affected region. We recommend harvest of tissue from unaffected areas if there is any question of perfusion. Finally, initial operative exploration represents a unique, favorable opportunity for exploration of recipient vessels especially in the acute phase and avoids the need for dissection in scarred tissue.^14^

The use of a dermal regeneration template such as Integra allows for immediate closure of the radial forearm donor site and coverage with skin graft in a single operation without the need for a second-stage skin graft at a second operation as previously described. Also, we propose that this method will avoid the potential hazards encountered by simply placing a skin graft in direct contact with exposed tendons and other structures. To our knowledge, we are documenting the first reported case of simultaneous Integra and split-thickness skin graft application at the initial operation without further intervention and facilitating graft take by NPWT. We plan to evaluate the efficacy of this method in a future case series.

A multistage, delayed reconstruction would have required, minimally, 2 more operations: (1) elevation of the flap with meticulous dissection through a scarred bed to identify the proximal and distal ends of the median nerve while preserving the reconstructed brachial artery, and (2) a typical second stage operation after 10 to 14 days of NPWT on the Integra dermal matrix for a skin graft. The combined benefits of a single operation as well as the potential for NPWT on an outpatient basis, as described by Leffler et al,^10^ may potentially lead to improvements in clinical outcome as well as decreases in overall operative time, length of hospital stay, and total cost to the patient. Future studies with larger series of patients are required to more accurately assess these outcomes.
